# Plant growth-promoting bacteria potentiate antifungal and plant-beneficial responses of *Trichoderma atroviride* by upregulating its effector functions

**DOI:** 10.1371/journal.pone.0301139

**Published:** 2024-03-22

**Authors:** Paulina Guzmán-Guzmán, Eduardo Valencia-Cantero, Gustavo Santoyo

**Affiliations:** Institute of Chemical and Biological Research, Universidad Michoacana de San Nicolás de Hidalgo, Morelia, Michoacán, México; Benemérita Universidad Autónoma de Puebla: Benemerita Universidad Autonoma de Puebla, MEXICO

## Abstract

*Trichoderma* uses different molecules to establish communication during its interactions with other organisms, such as effector proteins. Effectors modulate plant physiology to colonize plant roots or improve *Trichoderma*’s mycoparasitic capacity. In the soil, these fungi can establish relationships with plant growth–promoting bacteria (PGPBs), thus affecting their overall benefits on the plant or its fungal prey, and possibly, the role of effector proteins. The aim of this study was to determine the induction of *Trichoderma atroviride* gene expression coding for effector proteins during the interaction with different PGPBs, *Arabidopsis* or the phytopathogen *Fusarium brachygibbosum*, and to determine whether PGPBs potentiates the beneficial effects of *T*. *atroviride*. During the interaction with *F*. *brachygibbosum* and PGPBs, the effector coding genes *epl1*, *tatrx2* and *tacfem1* increased their expression, especially during the consortia with the bacteria. During the interaction of *T*. *atroviride* with the plant and PGPBs, the expression of *epl1* and *tatrx2* increased, mainly with the consortium formed with *Pseudomonas fluorescens* UM270, *Bacillus velezensis* AF12, or *B*. *halotolerans* AF23. Additionally, the consortium formed by *T*. *atroviride* and *R*. *badensis* SER3 stimulated *A*. *thaliana* PR1:GUS and LOX2:GUS for SA- and JA-mediated defence responses. Finally, the consortium of *T*. *atroviride* with SER3 was better at inhibiting pathogen growth, but the consortium of *T*. *atroviride* with UM270 was better at promoting *Arabidopsis* growth. These results showed that the biocontrol capacity and plant growth-promoting traits of *Trichoderma* spp. can be potentiated by PGPBs by stimulating its effector functions.

## Introduction

The rhizosphere is a zone inhabited by a myriad of different microorganisms associated with plants, known as the plant microbiome, and interactions among these organisms are constantly occurring, affecting their influence on the plants [1]. Interactions between microorganisms could result in synergistic effects on the plant, and the modulation of such a microbiome has been proven important for establishing more sustainable agricultural practices [2–4]. Several rhizosphere and plant endophyte inhabitants have been studied due to their ability to promote plant growth and protect plants against pathogens, such as fungi from the genus *Trichoderma* and plant growth–promoting bacteria (PGPB) [5,6].

*Trichoderma* spp. (Ascomycota, teleomorph: *Hypocrea*) are fungi that can protect plants by direct and indirect mechanisms, such as direct attack on the plant pathogen using secondary metabolites, mycoparasitism, or inducing plant defense responses and priming, traits that make these organisms excellent biocontrol agents [7,8]. In addition to their biocontrol capacity, *Trichoderma* spp. are used as biofertilizers owing to their ability to increase plant growth and yield [9,10]. Among the *Trichoderma* genus, *T*. *atroviride* is known for its capacity to increase plant growth and yield [11], and to antagonize several fungal plant pathogens, such as *Alternaria alternata*, *Botrytis cinerea*, *Rhizoctonia solani*, *Fusarium* spp., among others [12–14], induce plant defense response systems [15,16].

For *T*. *atroviride* to establish interactions with plants or pathogens, effector molecules mediate the molecular dialogue between these organisms, [17] and several studies have identified effector coding genes and molecules from *Trichoderma* [18–20]. This is the case for Epl1, a cerato-platanin protein that is considered a plant defence elicitor [21]. The expression of *epl1* in *Arabidopsis thaliana* protects the plant against pathogens such as *Botrytis cinerea* and *Pseudomonas syringae*, and accelerates plant growth [22]. Epl1 also modulates the expression of genes related to systemic acquired resistance (SAR) and induced systemic resistance (ISR) in tomato plants, protecting them against pathogens, such as *A*. *solani*, *B*. *cinerea* and *P*. *syringae* pv. *tomato* (*Pst* DC3000) [11].

Previously, we identified effector coding genes from *T*. *atroviride*, such as *tacfem1* and *tatarx2*, whose expression was induced either in response to the phytopathogen *R*. *solani* AG5 or the plant *A*. *thaliana* [23]. *tacfem1* increased its expression in the presence of *A*. *thaliana* upon contact with and overgrowth of plant roots, and it increased its expression during contact and overgrowth of *R*. *solani* AG5. The gene *tatrx2* showed increased expression when overgrowing the roots of *A*. *thaliana* [23], but its expression was not statistically significant when confronted with *R*. *solani* AG5 (data not published), suggesting that *tacfem1* and *tatrx2* are involved in *T*. *atroviride* interactions with both the plant and phytopathogen.

PGPB are widely used as biocontrol agents and biofertilizers because of their capacity to inhibit pathogen growth by producing secondary metabolites, volatile compounds, lytic enzymes, or lipopeptides. Additionally, PGPB can induce plant biomass and resistance against pathogens by eliciting the action of some metabolites and proteins excreted into the rhizosphere or plant roots [24–26]. Several species are considered beneficial to plants, including those belonging to genera such as *Pseudomonas* and *Bacillus*, which are among the heavy PGPBs [27–30].

For example, *Pseudomonas fluorescens* G20-18 induces salicylic acid-mediated defense in *A*. *thaliana* protecting the plant against *Pseudomonas syringae* [31] and promoting growth in cabbage plants [32,33]. Meanwhile, *Bacillus subtilis* induces resistance in potato plants against *Phytophthora infestans* [34], *Bacillus vallismortis* has antagonistic activity against the fungus *Macrophomina phaseolina* [35], and *Bacillus velezensis* VRU1 capsule formulation was shown to be effective against *Rhizoctonia solani* and protection against this pathogen[36]. Consortia formed with *Bacillus halotolerans* strains promoted *A*. *thaliana* and tomato growth and root system development and enhanced protection against *Botrytis cinerea* [37].

Both *T*. *atroviride* and PGPB have been widely used in agriculture to increase plant protection and yield. However, most studies have focused on the individual inoculation of either microorganism [24,30,38,39]. Currently, studies of microorganism consortia, including fungi and/or bacteria, have arisen because the interaction of beneficial microorganisms can have better benefits over agriculturally important crops [40–42]; however, the interactions among microorganisms forming consortia are still poorly understood.

Nonetheless, the benefits of using consortia are being studied and have proven to work better than the use of microorganisms alone in many cases. The consortium formed by *T*. *harzianum*, *B*. *amyloliquefaciens*, *B*. *subtilis* and *P*. *chlororaphis* proved to be effective in controlling *Fusarium oxysporum* disease in tomato plants and is better than microorganism inoculation alone [43]. The combination of two *Triochoderma* spp. strains, BHU51 and BHU105, had better results in diminishing disease symptoms in *Solanum melongena* caused by *Sclerotium rolfsii* than inoculation with the strains alone, also increasing plant growth [44]. The consortium formed by *Trichoderma lixii* and *Streptomyces atrovirens* reduced disease symptoms in tomato plants caused by *R*. *solani* and induced plant resistance against the phytopathogen [45]compared to the effect of the microorganisms alone.

Studies have highlighted the importance of effectors during interactions between microorganisms as a form of molecular dialogue to establish interactions [17,46,47], such as those between *Trichoderma* spp. and plants. Nonetheless, the role of effectors in the interactions of *Trichoderma* with other beneficial microbes, such as beneficial bacteria, is unknown. Therefore, the aim of this study was to determine the expression of the *T*. *atroviride* effector coding genes *epl1*, *tatrx2* and *tacfem1* during interactions with different PGPBs, *A*. *thaliana* plants, or the phytopathogen *Fusarium brachygibbosum* and to determine whether the combined interaction potentiates the beneficial effects of *T*. *atroviride* (and PGPB) on the plant and against the fungal pathogen.

## Materials and methods

### Organisms’ growth and culture conditions

PGPB *Rouxiella badensis* SER3 (SER3) [48], *Pseudomonas fluorescens* UM270 (UM270) [49], *Bacillus velezensis* AF12 (AF12), and *Bacillus halotolerans* AF23 (AF23) [50] were grown in nutrient broth medium (NB) for 24h at 30°C. Dilutions of each liquid culture were prepared and plated on Petri dishes containing nutrient agar medium (NA), incubated overnight at 30°C, and then bacterial colonies were counted to determine the CFU of each PGPB and adjusted to a concentration of 1x10^6^ CFU/mL for the experiments. *Trichoderma atroviride* IMI206040 (Ta) [51] was inoculated in Petri dishes containing potato dextrose agar medium (PDA) and kept at 28°C in darkness until full conidiation (7–10 days), collected with sterile distilled water, filtered using Magitel ® filters, and adjusted to a concentration of 1x10^6^ conidia/mL for the experiments. *Fusarium brachygibbosum* [48] was inoculated in Petri dishes with PDA and incubated at 28°C in darkness until full coverage of the plate to obtain mycelia actively growing for the experiments. Fresh cultures were prepared before any experiments were conducted, and stock cultures were maintained at 4°C. *Arabidopsis thaliana* Col– 0 (At) and *A*. *thaliana* kanamycin-resistant transgenic lines PR1:GUS and LOX2:GUS [52] seeds were sterilized with 96% ethanol five times, air dried in a laminar flow chamber until completely dry, and kept in sterile conditions until use. The seeds were vernalized for 48h at 4°C in the dark and then germinated in Murashige–Skoog medium (MS) in a plant growth chamber (16h light/8h darkness at 22°C). Transgenic seed lines were vernalized for 48h at 4°C in the dark and then germinated in MS with 50μg/mL of kanamycin.

### Consortia formed by *T*. *atroviride* and PGPBs

We used the following combinations of *T*. *atroviride* with each PGPB: *T*. *atroviride* + *R*. *badensis* SER3 (Ta+SER3), *T*. *atroviride* + *P*. *fluorescens* UM270 (Ta+UM270), *T*. *atroviride + B*. *velezensis* AF12 (Ta+AF12) and *T*. *atroviride* + *B*. *halotolerans* AF23 (Ta+AF23).

### Co–inoculation conditions of *T*. *atroviride* and PGPBs

We first tested whether both types of microorganisms could grow together in the medium used for the experiments. Petri dishes containing PDA medium were inoculated with each PGPB strain to form a cross, thereby dividing the plate into four quadrants. Plugs of actively growing mycelia and 1x10^6^ conidia from *T*. *atroviride* were inoculated in each quadrant (S1 Fig). Plates were incubated at 30°C in darkness for 72h and then checked to determine whether bacteria and *T*. *atroviride* could grow together and conidial germination would not be inhibited. We repeated the experiment using the MS 0.2X medium, and each experiment was performed three times with similar results.

### *T*. *atroviride–*PGPB biocontrol experiments

Biocontrol experiments were performed using PDA medium. Plugs of actively growing mycelia from *T*. *atroviride* or *F*. *brachygibossum* were placed on opposite sides of the petri plate, and the corresponding bacterial strain was striated opposite from both mycelial plugs to test the effect of the consortia on pathogen growth. Plugs containing the pathogen alone were used as controls. Inoculated plates were incubated at 28°C in the dark for 3, 5, and 7 days, at which time photographs of each treatment were taken. Three biological experiments were conducted in triplicates for each treatment. Photographs were analyzed using ImageJ [53] software to determine the area of pathogen growth in each treatment. The percentage growth inhibition was calculated using the following formula:

$$\%Growth\;Inhibition=\frac{{Fb}_{control}-{Fb}_{treatment}}{{Fb}_{treatment}}\times 100$$

Where Fb_control_ is the area of the pathogen growing alone and Fb_treatment_ is the area of the pathogen co-inoculated with the microorganisms alone or in the consortium.

### *T*. *atroviride–*PGPB plant growth promotion experiments

Plant interaction experiments were conducted in MS 0.2 × supplemented with MES as a pH buffer at a concentration of 1 g/L [54]. Ten sterilized *A*. *thaliana* seeds were placed on one side of the plate and vernalized in the darkness for 48h at 4°C. Seeds were placed in a plant growth chamber (16h light/8h darkness and 22°C) to germinate, and on the 4^th^ day of germination, the microorganisms alone or in consortium were inoculated. 1x10^6^ conidia from *T*. *atroviride* were inoculated approximately 5 cm from the plant roots, and the corresponding PGPB (1x10^6^ CFU) was striated below the *Trichoderma* inoculum. Interactions were conducted for three and five days. Primary root length, number of lateral roots, plant height, and fresh and dry weight data were taken at the 3^rd^ and 5^th^ day post inoculation (dpi). Plants growing axenically were used as controls.

### *T*. *atroviride–*PGPB plant defense systems induction

*A*. *thaliana* kanamycin-resistant transgenic lines PR1:GUS and LOX2:GUS were used to evaluate the induction of plant defense systems by the microorganisms alone and in the consortium. In the PR1:GUS transgenic line, the promoter from the *Arabidospis* gene *pr-1* is merged with the reporter gene GUS (β-glucuronidase) in response to salicylic acid (SA) accumulation [55,56], while the promoter from *Arabidopsis lox2* gene is merged with the reporter gene GUS, responding to jasmonic acid (JA) accumulation in the plant [57,58]. Interactions between PR1:GUS or LOX2:GUS and microorganisms and consortia were conducted as described above. After 5 days of interaction, plants were incubated with the reagent X-Gluc at 37°C in total darkness for 24 h to promote the reaction and precipitation of the insoluble compound 5,5’-dibromo-4,4’dichloro-indigo, shown in blue in the plant tissue [59]. After incubation, the plants were washed with ethanol to eliminate chlorophyll and rehydrated with 50% ethanol/ 50% glycerol. Cleared plants were placed on microscope slides for observation under a stereomicroscope and photographed. Thirty plants per treatment were examined and photographed.

### *T*. *atroviride* effector coding genes relative expression

To collect mycelia from *T*. *atroviride* during interactions with PGPBs and in confrontation with either *A*. *thaliana* or *F*. *brachygibbosum*, interactions were conducted as mentioned above, but Petri dishes were covered with a sterile cellophane membrane prior to inoculation or seed placement. Mycelia from the front of the actively growing colony of *T*. *atroviride* (0.5 cm) were placed inside a microcentrifuge tube, immediately frozen in liquid nitrogen, and stored at -80°C until processing. Total RNA was extracted from frozen samples following the protocol for the TriZol ® method, and RNA integrity was verified by agarose gel electrophoresis and quantified using a Nanodrop (Thermo Scientific). cDNA was synthesized using GoScript ™ Reverse Transcriptase (Promega) according to the manufacturer’s protocol and quantified using a Nanodrop. qRT-PCR was carried out with SybrGreen master mix (Radiant ™) using 20ng of cDNA as a template. The glyceraldehyde-3-phosphatedehydrogenase gene (*gpd*) was used as the housekeeping gene, and three technical replicates from each sample were analyzed. The ΔΔCt method was used to determine the relative expression of the genes from the qRT-PCR assays using StepOne software (Applied Biosystems). The primers used in this study were previously designed [23] and are described in S1 Table.

### Statistical analyses

One-way ANOVA with Tukey’s *post hoc* test was used to analyze the results from the biocontrol experiments. Two-way ANOVA with Dunnett’s *post hoc* test of mean comparison was used to analyze the results from the plant growth-promotion and relative expression experiments. GraphPad Prism 8 software (GraphPad Software, Inc.) was used for the statistical analyses.

## Results and discussion

### Results

#### Compatibility of the consortia *T*. *atroviride–*PGPB

**Fig 1:**
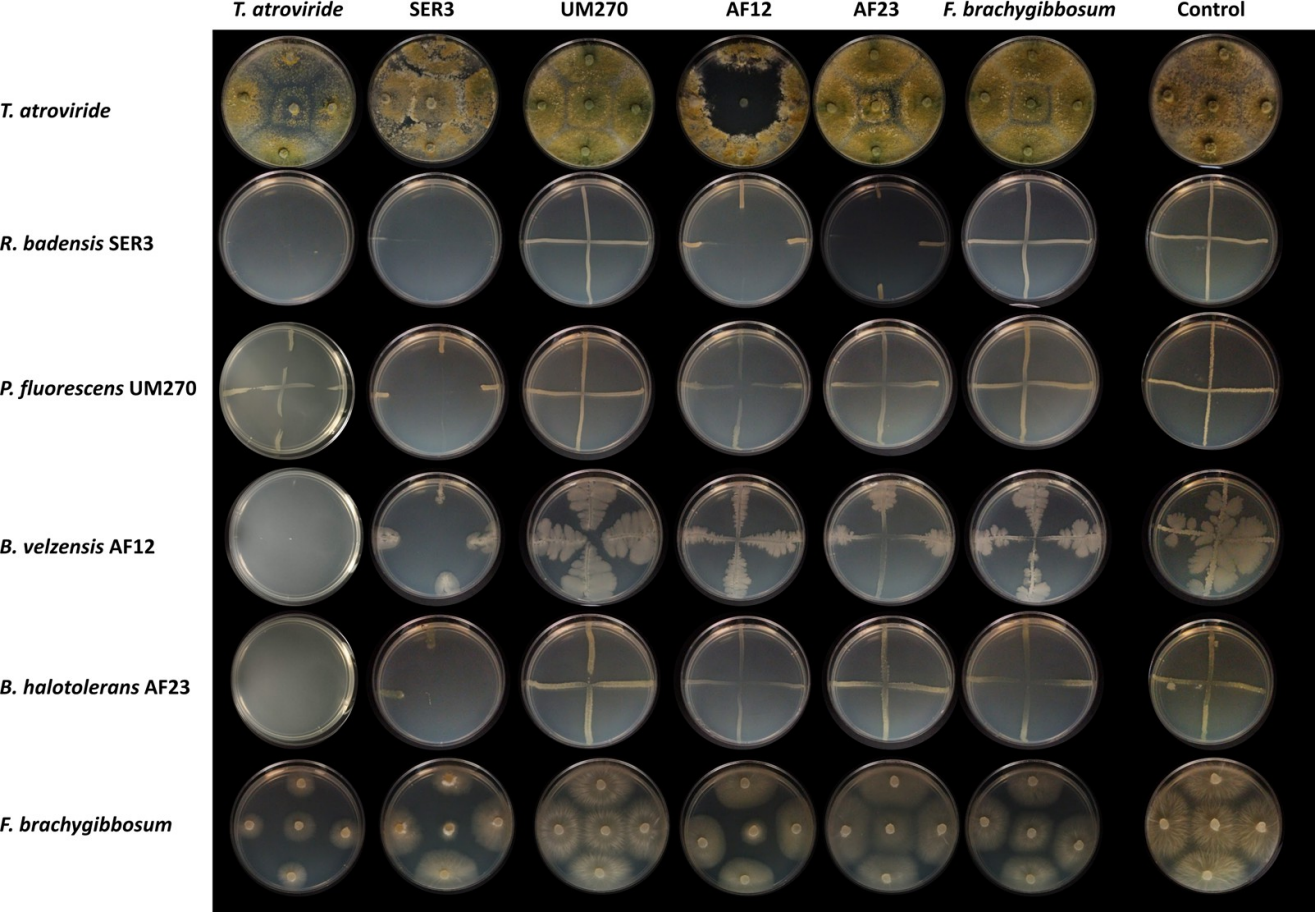
Inhibition of microorganisms by secreted compounds. Each PGPB, *T*. *atroviride* and *F*. *brachygibbosum* were inoculated over cellophane in Petri dishes containing PDA medium, and after 3 days, cellophane and the growing microorganism were removed (names at the top of the image indicate the secreted compounds left in the medium by the microorganisms). Each PGPB, *T*. *atroviride* and *F*. *brachygibbosum* were then inoculated over the dishes without the cellophane, and their growth was observed at 7th dpi (names at the left of the image indicate the microorganism growing on the Petri dish). Control condition is each microorganism growing alone without secreted compounds in the medium. Photographs are representative of two independent experiments with 3 individual replicates.

Since the goal of this work was to use the consortium *T*. *atroviride* and PGPBs, we first attempted to grow both organisms in the media to be used during the interactions. We co–inoculated *T*. *atroviride* conidia and mycelia with each of the four PGPBs used in this study in PDA and MS 0.2 × media. The results showed that *T*. *atroviride* can germinate from conidia and actively grow in the presence of each PGPB tested, and that each PGPB was able to grow in the presence of *T*. *atroviride* in both PDA and MS media (Fig 1). Additionally, we decided to perform an experiment, as indicated by Prigallo et al.(2022), to observe whether each of the microorganisms’ secreted compounds could inhibit each other and help with the selection of the best consortium or consortia. In general, our results show that beneficial microorganisms can inhibit each other and, on occasion, can even inhibit themselves, as in the case of SER3, which showed strong inhibition. This also indicates that *T*. *atroviride* can grow with any microorganism but can inhibit other beneficial microorganisms (except for UM270), and we confirmed that *B*. *velezensis* AF12 could inhibit *T*. *atroviride* (Fig 1).

#### Pathogen’s growth inhibition ability of the consortia *T*. *atroviride–*PGPB

**Fig 2:**
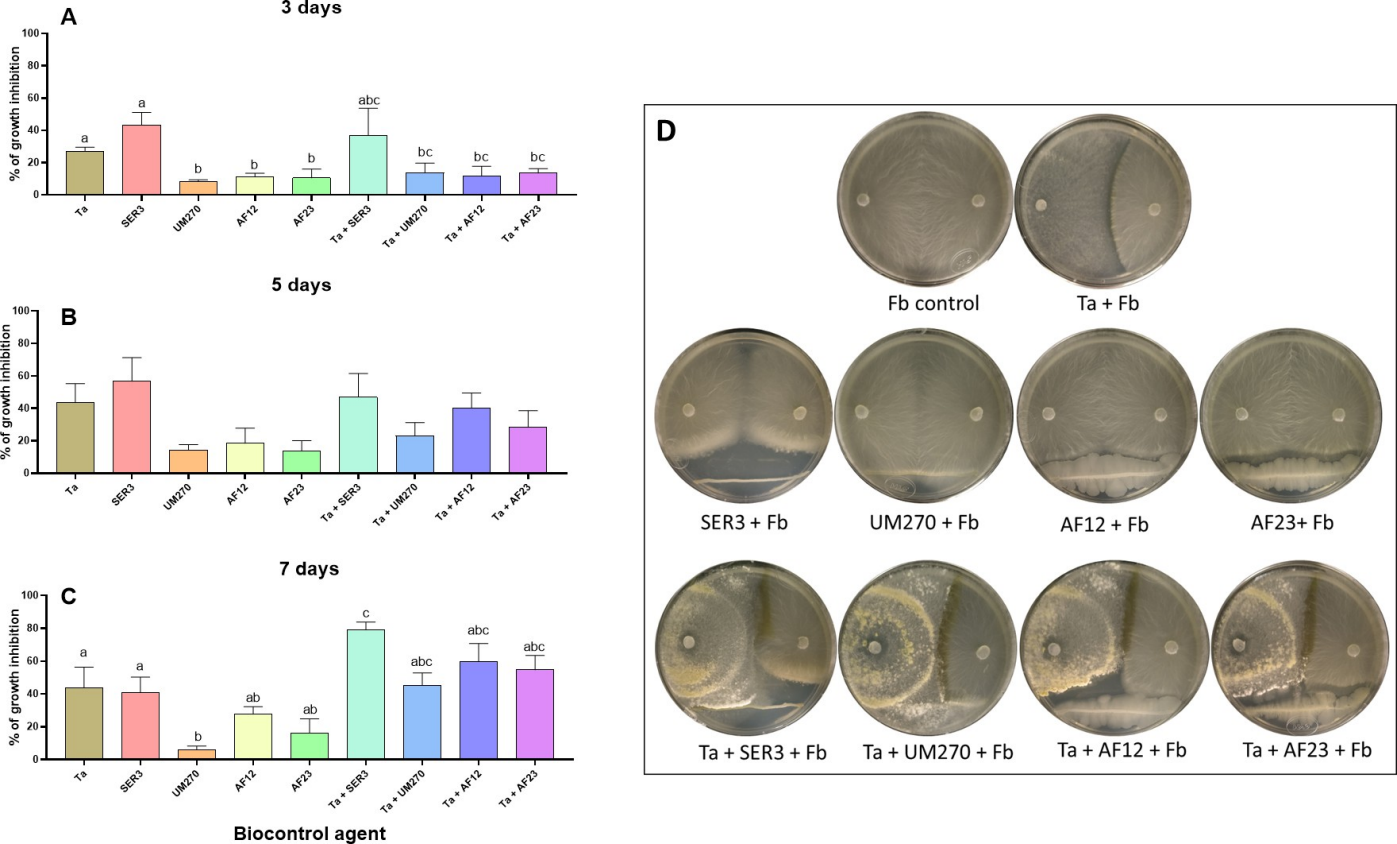
*Fusarium brachygibbosum* growth inhibition. Growth inhibition of the phytopathogen *F*. *brachygibbosum* (Fb) in confrontation with each BCA and consortia at 3, 5 and 7 days of interaction (A, B and C, respectively). D), Representative photographs of the experiment at the 7^th^ day of confrontation. One-way ANOVA with Tukey *post hoc* test for comparison of treatments; different letters above bars indicate statistical significance of *p<*0.05; bars indicate SEM. Fb, *F*. *brachygibbosum;* Ta, *T*. *atroviride*; SER3, *R*. *badensis* SER3; UM270, *P*. *fluorescens* UM270; AF12, *B*. *velezensis* AF12; AF23, *B*. *halotolerans* AF23.

Being verified that the microorganisms can grow in each other presence, we proceeded to determine their ability to inhibit the growth of the phytopathogen *F*. *brachygibbosum*, which was found to be a postharvest pathogen [48]. Confrontations were carried out as indicated in the methodology section, and at 3, 5, and 7 days of interaction, photographs of each Petri dish were taken to measure the area of growth of the pathogen. Fig 2D shows representative photographs of the experiment at the 7^th^ day of the confrontation.

On the 3^rd^ day of confrontation, the biocontrol agents alone showed the following inhibition percentages of the phytopathogen *F*. *brachygibbosum*: (Ta) inhibited pathogen growth by 27.22%, SER3 showed 43.39% inhibition, *P*. *fluorescens* UM270 inhibited the pathogen only at 8.36%, AF12 inhibited 11.41%, and *B*. *halotolerans* AF23 inhibited 10.55% (Fig 2A). The biocontrol agents in the consortium showed the following percentages of inhibition on the 3^rd^ day of confrontation: Ta+SER3 inhibited the growth of *F*. *brachygibbosum* by 36.97%, Ta+UM270 inhibited the pathogen by 13.825%, Ta+AF12 inhibited 8.9%, and Ta+AF23 inhibited 13.57% (Fig 2A). These results show that the capacity of *T*. *atroviride* to inhibit the pathogen increased during the early stages of the confrontation in the presence of *R*. *badensis* SER3, and the ability to inhibit the phytopathogen of *P*. *fluorescens* UM270, *B*. *velzensis* AF12, and *B*. *halotolerans* AF23 slightly increased in the presence of Ta, but no statistically significant differences were found among these treatments (Fig 2A).

**Fig 3:**
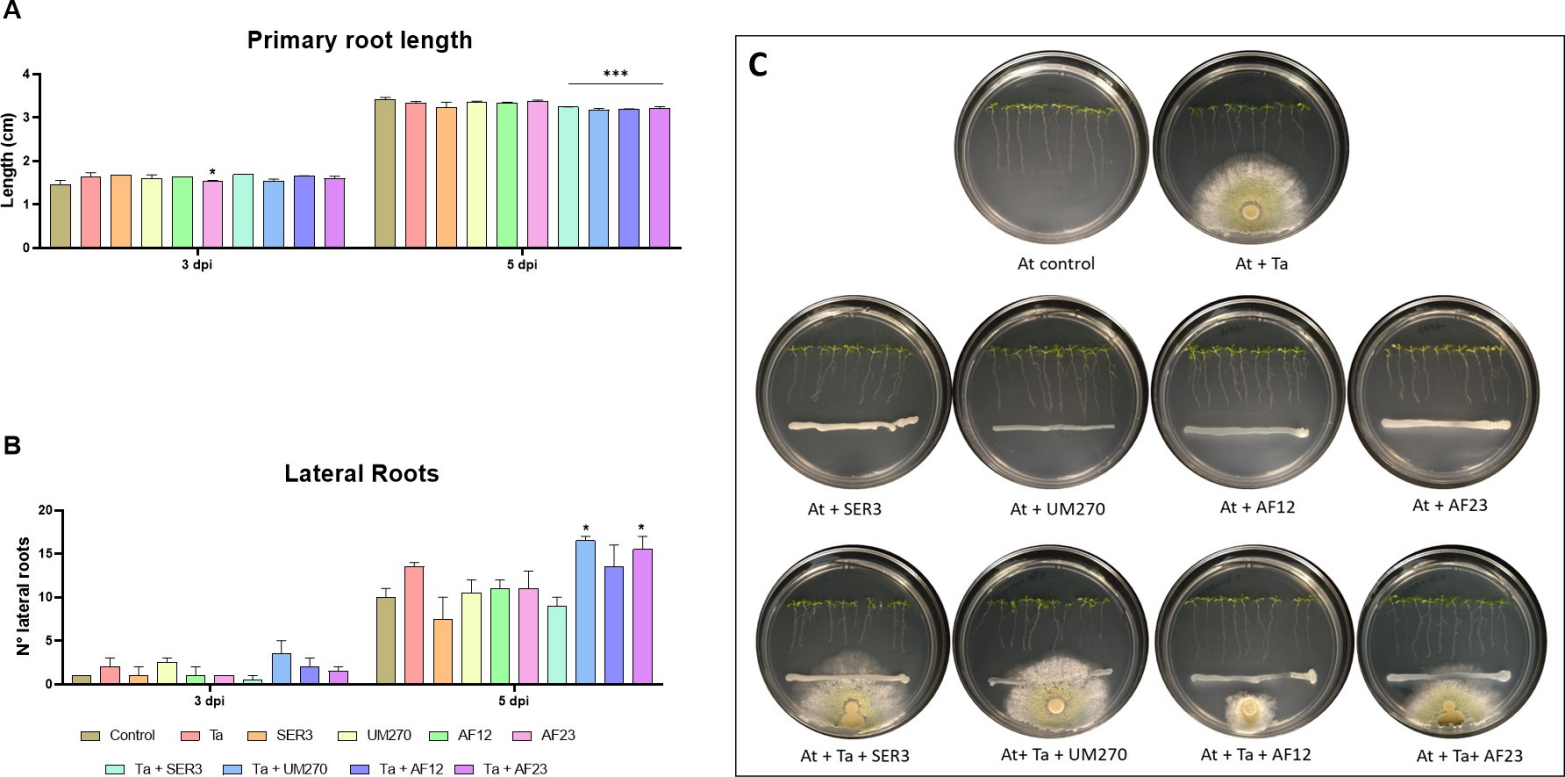
Plant growth promotion of *A*. *thaliana*. Effect of the co–inoculation of the different biostimulants and consortia on main root length (A) and number of lateral roots (B). (C) Representative photographs of the experiment at the 5^th^ day of interaction. At, *A*. *thaliana;* Ta, *T*. *atroviride*; SER3, *R*. *badensis* SER3; UM270, *P*. *fluorescens* UM270; AF12, *B*. *velezensis* AF12; AF23, *B*. *halotolerans* AF23. Two-way ANOVA with Dunnet *post hoc* test of mean comparison, *p<0.05, **p<0.01, ***p<0.001 compared to control.

At the 5^th^ day of confrontation, Ta inhibited *F*. *brachygibossum* growth in 43.55%, SER3 showed a 56.64% of inhibition, UM270 inhibited the pathogen in 14.34%, AF12 in 18.42% and AF23 inhibited *F*. *brachygibbosum* in 13.69%; the consortium Ta+SER3 inhibited the growth of *F*. *brachygibbosum* in 46.99%, Ta+UM270 showed 23.07%, and the consortia Ta+AF12 and Ta+AF23 inhibited the pathogen growth in 40.08% and 28.30%, respectively, but no statistically significant differences were found among all treatments (Fig 3A). Despite no statistically significant differences at this stage, there was a tendency for the inhibition ability of Ta to increase in the presence of SER3, and the inhibition capacity of UM270, AF12, and AF23 increased in the presence of Ta.

At the end of the experiment (7^th^ day of confrontation), we can see in Fig 2C that Ta inhibited the pathogen growth by 43.77%, SER3 inhibited *F*. *brachygibbosum* by 41.06%, UM270 inhibited the pathogen by 6.16%, and AF12 and AF23 inhibited phytopathogen growth by 27.65% and 16.05%, respectively. The consortia biocontrol agents showed increased percentage of inhibition against *F*. *brachygibbosum*, compared to the biocontrol agents alone; Ta+UM270 inhibited the pathogen in 45.43%, Ta+AF12 in 59.86% and Ta+AF23 in 54.67%, but the consortium Ta+SER3 inhibited the growth of *F*. *brachygibbosum* in 78.96% (Fig 2C), being statistically different than the organisms alone against the pathogen. These results clearly show that the consortium formed by *T*. *atroviride* and the PGPB *R*. *badensis* SER3 was better at inhibiting the growth of the pathogen than the organisms alone, and the presence of *T*. *atroviride* increased the capacity of the other PGPBs tested to inhibit the pathogen growth.

#### Plant growth–promoting ability of the consortia *T*. *atroviride–*PGPB

Plant growth–promoting bacteria and *T*. *atroviride* are excellent microorganisms used as biostimulants and biofertilizers because of their ability to improve plant growth, development, and yield, and increase plant biomass and root system [5,10]. We performed interaction assays between the organisms alone and in combination with *A*. *thaliana*, and on the 3^rd^ and 5^th^ day of interaction, measures of primary root length, number of lateral roots, plant height, and fresh and dry weight were measured. Fig 3C shows representative photographs of the experiment at the 5^th^ day of the interaction.

With regard to the primary root length of *Arabidopsis* plants, on the 3^rd^ day of interaction, primary roots of plants growing in the presence of AF23 were shorter than those of the control plants and those of the other treatments (*p*<0.05; Fig 3A). On the 5^th^ day of interaction, roots of plants growing in the presence of all the consortia were shorter than those of the control plants and plants growing with the organisms alone (*p*<0.001; Fig 3A). On the 3^rd^ day of interaction, we found no statistically significant differences in the number of lateral roots among the treatments (*p>* 0.05; Fig 3B); nonetheless, on the 5^th^ day of interaction, plants growing in the presence of the consortia Ta+UM270 and Ta+AF23 had significantly more lateral roots than control plants and that of the plants growing with the microorganisms alone, 65% and 55%, respectively, more than control plants (*p*<0.05; Fig 3B). These results show that the consortia formed by *T*. *atroviride* and the PGPBs *P*. *fluorescens* UM270 or *B*. *halotolerans* AF23 are better at developing the secondary root system of *A*. *thaliana* plants than organisms growing alone.

**Fig 4:**
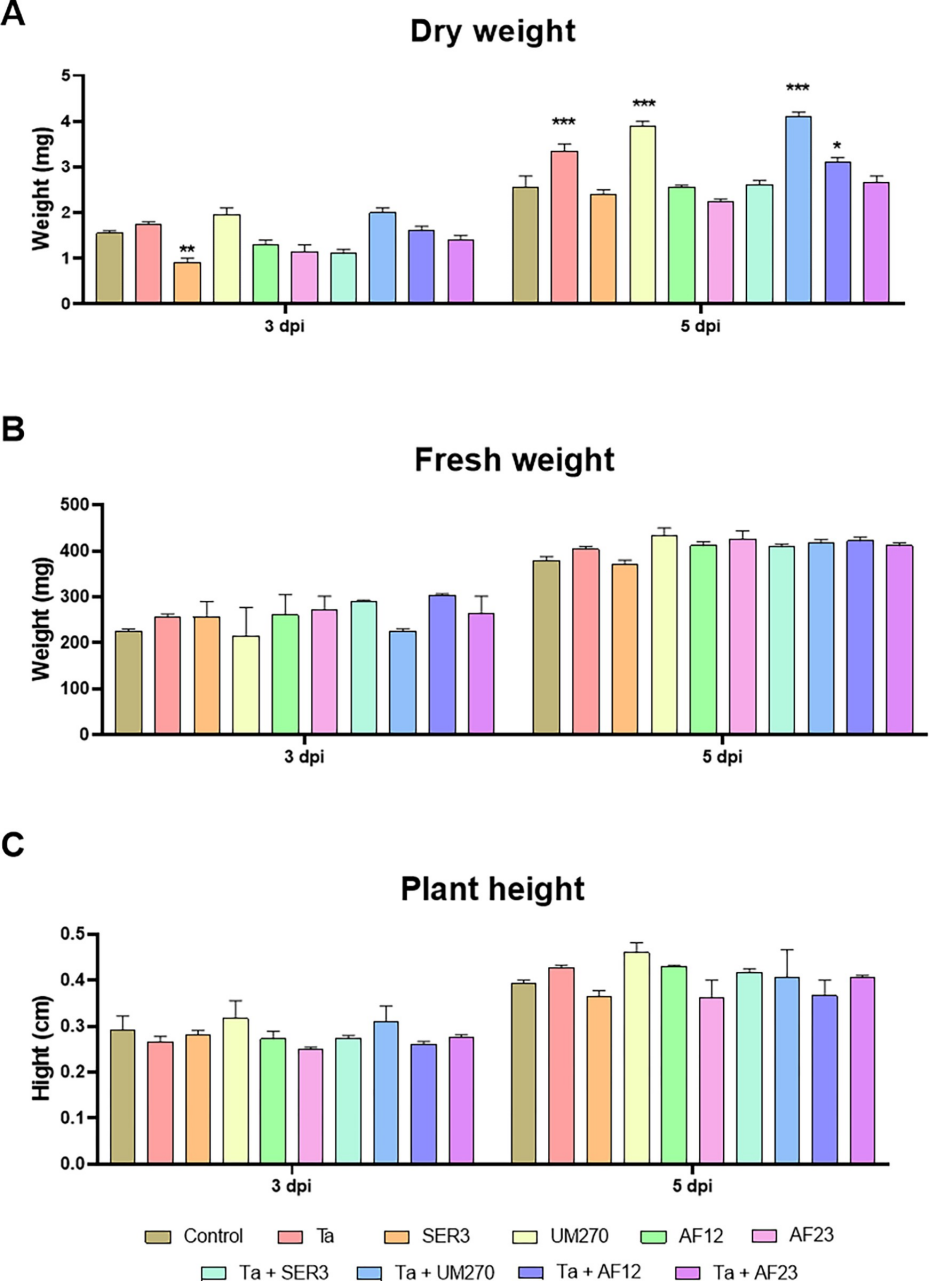
Biomass of *A*. *thaliana*. Effect of the co–inoculation of the different biostimulants and consortia on plant dry weight (A), fresh weight (B) and plant height (C). Two-way ANOVA with Dunnet *post hoc* test of mean comparison, *p<0.05, **p<0.01, ***p<0.001 compared to control.

Regarding the dry weight of *Arabidopsis* plants, plants growing in the interaction with SER3 weighed 41.9% less than that of the control plants (*p*<0.01; Fig 4A), and no statistically significant differences were found between control plants and the other treatments tested for this experiment (*p>* 0.05; Fig 4A) on the 3^rd^ day of interaction. However, on the 5^th^ day of interaction, plants growing in the interaction with Ta, UM270, and the consortia Ta+UM270 and Ta+AF12 had more dry weight than the control plants, with 31.4%, 52.9%, and 60.8% (*p<* 0.001; Fig 4A) and 21.5% (*p<* 0.05; Fig 4A), respectively. We found no statistically significant differences between the treatments and control plants regarding plant fresh weight and plant height (*p>* 0.05; Fig 4B and 4C, respectively), although plants growing in the presence of Ta, UM270, and the consortium Ta+UM270 were slightly larger than the control plants at the end of the experiment. These results indicate that not only *T*. *atroviride* and *P*. *fluorescens* UM270 good microorganisms for increasing plant biomass, but their consortium is better than both, increasing dry weight by 60.78% %over plants growing alone; thus, the consortium formed by *T*. *atroviride* and *P*. *fluorescens* UM270 could be a better option as a biostimulant for increasing plant biomass.

#### Expression of effector coding genes of *Trichoderma* during the mycoparasitic interaction

Effector proteins are molecules that mediate communication between microorganisms such as *Trichoderma* and plants or their fungal prey [17,47]. We determined the expression of three effector-coding genes from *T*. *atroviride*: *epl1*, *tatrx2* and *tacfem1*, during its interaction with PGPBs and the phytopathogen *F*. *brachygibbosum*. The experiment was performed as described in the methodology section. Mycelia from *T*. *atroviride* were collected from confrontations between *T*. *atroviride* and each PGPB and the consortia *T*. *atroviride*–PGPB with the phytopathogen. RNA extraction and cDNA synthesis were carried out to determine gene expression by qRT-PCR, and the fungus growing alone served as the control.

**Fig 5:**
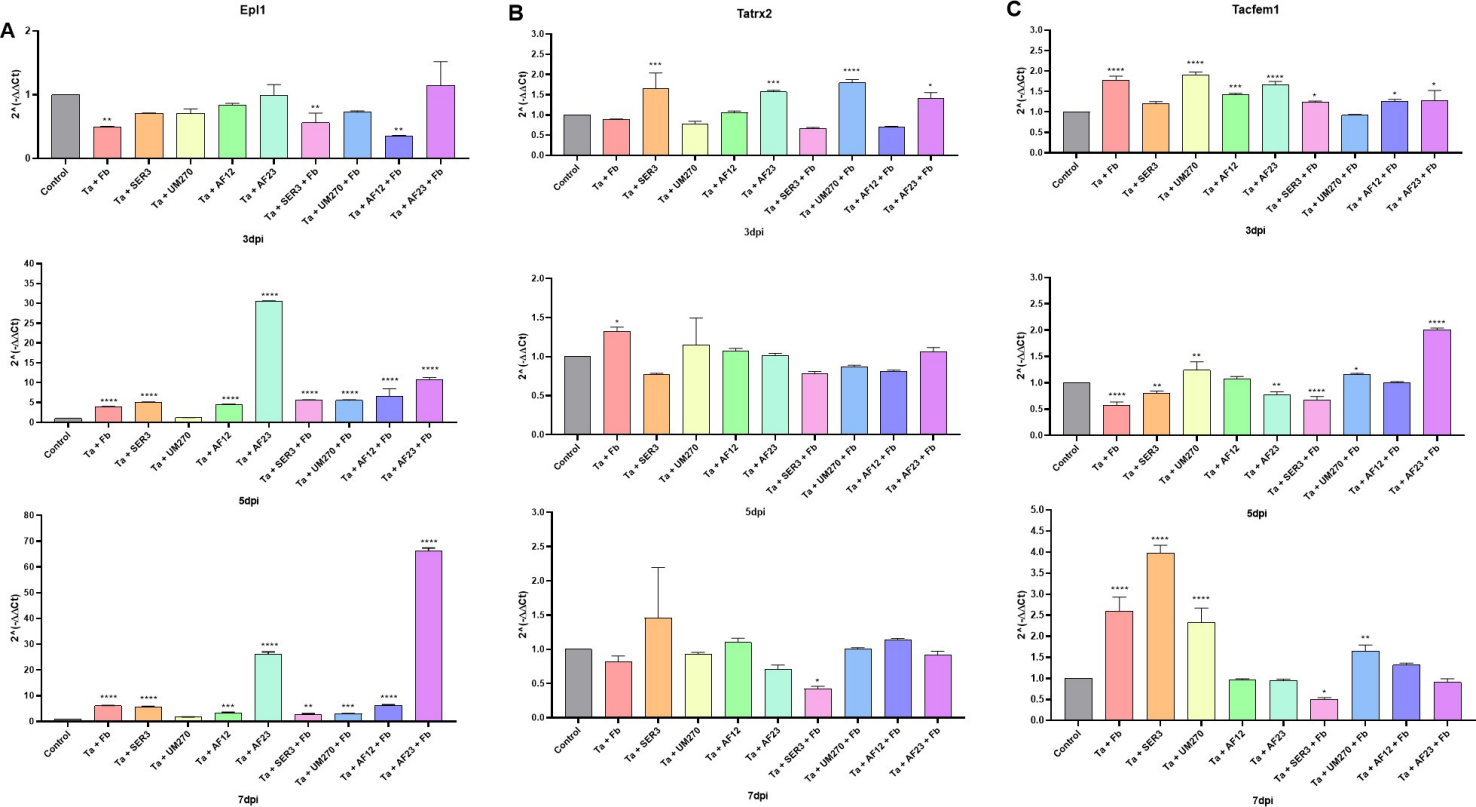
Relative gene expression of *Trichoderma* effectors in the mycoparasitic interaction. Gene expression of *T*. *atroviride’*s effector coding genes *epl1* (A), *tatrx2* (B) and *tacfem1* (C) in the mycoparasitic interaction with Fb and PGPBs. One-way ANOVA with Dunnet *post hoc* test of mean comparison, *p<0.05, **p<0.01, ***p<0.001, ****p<0.0001 compared to control conditions (fungus growing alone).

Epl1 is a member of a protein family that is known to be an elicitor of plant defenses [11,21]. Nonetheless, the expression of *epl1* from *T*. *asperellum* was induced in the presence of mycelial powder and fermentation liquid from the fungal pathogen *Alternaria alternata* after 72h of exposure [60]. In our experiment, on the 3^rd^ day of confrontation, *epl1* was not induced in any of the treatments, compared to control conditions (*p>* 0.05), that is, the fungus grew alone, but it was downregulated in the presence of phytopathogen, phytopathogen, SER3, phytopathogen, and AF12 (*p<* 0.01; Fig 5A, upper graph).

On the 5^th^ day of confrontation, *epl1* was upregulated when *T*. *atroviride* was present in the presence of every microorganism (*p<*0.001; Fig 5A middle graph), except in the presence of UM270, where there was no statistically significant difference compared to the control (1.15 ± 0.02; *p>*0.05; Fig 5A middle graph). *epl1* is upregulated in the presence of the phytopathogen (3.91 ± 0.08 fold increase, *p*<0.001), in the presence of SER3 and AF12 (5.07 ± 0.05, 4.51 ± 0.06 fold increase, respectively; *p*<0.001), and *epl1* is also upregulated in the presence of Fb and SER3, Fb and UM270 and Fb and AF12 (5.62 ± 0.05, 5.54 ± 0.10, 6.66 ± 1.05 fold increase respectively; *p*<0.001) compared to the fungus growing alone. It is worth noting that *epl1* showed the most increase in its expression in the presence of AF23 with 30.58 ± 0.05 (*p*<0.0001) fold increase, and in the presence of both AF23 and Fb, showing a 10.79 ± 0.33 fold increase (*p*<0.0001) compared to control conditions (Fig 5A middle graph).

On the 7^th^ day of confrontation (Fig 5A, lower graph), *epl1* showed no induction in the presence of UM270 compared to the control (1.69 ± 0.03; *p*>0.05). In the presence of the pathogen Fb and PGPB SER3, *epl1* was induced (5.63 ± 0.12-and 6.05 ± 0.08 fold increase, respectively; *p<*0.0001), and this gene was also induced in the presence of AF12 (3.33 ± 0.14 fold increase; *p<*0.001) compared to the fungus growing alone. *Epl1* expression was induced in the presence of Fb and SER3 (2.71 ± 0.20; *p<*0.01), Fb and UM270 (3.0 ± 0.03; *p<*0.001), and Fb and AF12 (6.28 ± 0.14; *p<*0.0001). In the presence of AF23 and both Fb and AF23, *epl1* showed the highest increase in expression compared to the fungus growing alone, with 26.12 ± 0.51 and 66.20 ± 0.68 fold increase, respectively (*p<*0.001). These results show that Epl1 could play a role in mediating communication with beneficial bacteria, especially *B*. *halotolerans* AF23, and in mycoparasitic interactions with *F*. *brachygubbosum*.

Thioredoxin proteins, such as Tatrx2, are important for several fungal processes, including growth and oxidative stress tolerance [61–63], and mediate symbiosis establishment in legume plants [64]. On the 3^rd^ day of confrontation (Fig 5B, upper graph), the gene *tatrx2* was induced in the presence of SER3 (1.65 ± 0.23 fold increase; *p<* 0.001) and AF23 (1.57 ± 0.02; *p<* 0.001), with the presence of both UM270 + Fb (1.79 ± 0.05; *p<* 0.001), and it was also induced in the presence of AF23 + Fb (1.40 ± 0.09; *p<* 0.05) in comparison to control conditions. We found no statistically significant differences between the other treatments and the fungus growing alone (*p>*0.05; Fig 5B upper graph). At the 5^th^ day of confrontation (Fig 5B middle graph), none of the treatments induced *tatrx2* expression compared to control conditions (*p>*0.05), except for the pathogen, were *tatrx2* is 1.32 ± 0.03 fold increase (*p<*0.05) compared to the fungus growing alone. On the 7^th^ day of confrontation (Fig 5B, lower graph), there were no statistically significant differences in the expression of *tatrx2* in the treatments, compared to the fungus growing alone (*p>*0.05), except for SER3+Fb, where this gene was downregulated compared to the control (0.42 ± 0.02 fold increase; *p<*0.05). These results suggest that Tatrx2 may be mainly involved at the beginning of the confrontations, especially in the presence of *R*. *badensis* SER3 and in the presence of both *P*. *fluorescens* UM270 and *F*. *brachygibbosum*.

Regarding the expression of the gene *tacfem1*, this gene is a membrane receptor [65] and its expression is induced during the interaction of *T*. *atroviride* with the phytopathogen *R*. *solani* AG5 [23], and in general, CFEM effectors have a role in fungal pathogenicity [66]. In this study, on the 3^rd^ day of confrontation (Fig 5C upper graph), *tacfem1* was induced in the presence of Fb, UM270, AF23 (1.77 ± 0.05, 1.91 ± 0.04, 1.67 ± 0.04 fold increase, respectively; *p<*0.001), and in the presence of AF12 (1.43 ± 0.02 fold increase; *p<*0.001), compared to control conditions. In confrontation with both pathogen and PGPB, *tacfem1* was induced in the presence of SER3+Fb, AF12+Fb, and AF23+Fb (1.24 ± 0.02, 1.26 ± 0.02, 1.27 ± 0.15 fold increase respectively; *p<*0.05) compared to the fungus growing alone. We found no significant differences between the other treatment groups and the control group (*p>*0.05). On the 5^th^ day of confrontation (Fig 5C middle graph), *tacfem1* was downregulated compared to the control in the presence of Fb (0.58 ± 0.03; *p<* 0.001), SER3, AF23 (0.78 ± 0.02, 0.78 ± 0.03, respectively; *p<*0.01), and both SER3 and Fb (0.68 ± 0.04; *p<*0.001). However, *tacfem1* was upregulated in the presence of UM270 (1.24 ± 0.09; *p<*0.01), UM270+Fb (1.16 ± 0.01; *p<*0.05), and AF23+Fb, where its expression was induced by 2.01 ± 0.02 fold increase (*p<*0.0001) compared to the fungus growing alone. On the 7^th^ day of confrontation (Fig 5C, lower graph), the gene *tacfem1* was induced when *T*. *atroviride* was in the presence of Fb, SER3, and UM270 (2.59 ± 0.19, 3.98 ± 0.11, 2.33 ± 0.19 fold increase, respectively; *p<*0.0001), and in the presence of both UM270+Fb (1.65 ± 0.08 fold increase; *p<*0.01) compared to the fungus growing alone. *Tacfem1* was downregulated in the presence of SER3+Fb (0.51 ± 0.02; *p<*0.05) compared to the corresponding control. These results indicate that Tacfem1’s main role could be at the end of the confrontation, where *Trichoderma* overgrows the pathogen and is in contact with PGPBs, specifically with *R*. *badensis* SER3 and *P*. *fluorescens* UM270.

Altogether, the results of the expression of effector coding genes from *T*. *atroviride* in interaction with PGPBs and the phytopathogen *F*. *brachygibbosum* suggest that the effectors selected are involved at different stages of the confrontation, their induced expression depends on the microorganisms in question, and they respond to the presence of other beneficial microorganisms such as the PGPBs used in this work.

#### Expression of effector coding genes of *Trichoderma* during the beneficial interaction

One of the main studies on *T*. *atroviride* is its beneficial relationship with different plants, such as *A*. *thaliana*. *Trichoderma* uses effector molecules to mediate communication between its hosts, like Epl1, Tatrx2 and Tacfem1. However, the role of these effectors in interactions with other microorganisms, such as PGPBs and plants, is not known. We evaluated the expression pattern of these effector coding genes during the interaction with *A*. *thaliana* and the PGPBs used in this study at two time points: before contact with the plant (3^rd^ dpi) and at initiation of contact of the fungus with the plant roots (5^th^ dpi), in order to elucidate the possible role of these effectors in interactions with other plant-beneficial microbes.

**Fig 6:**
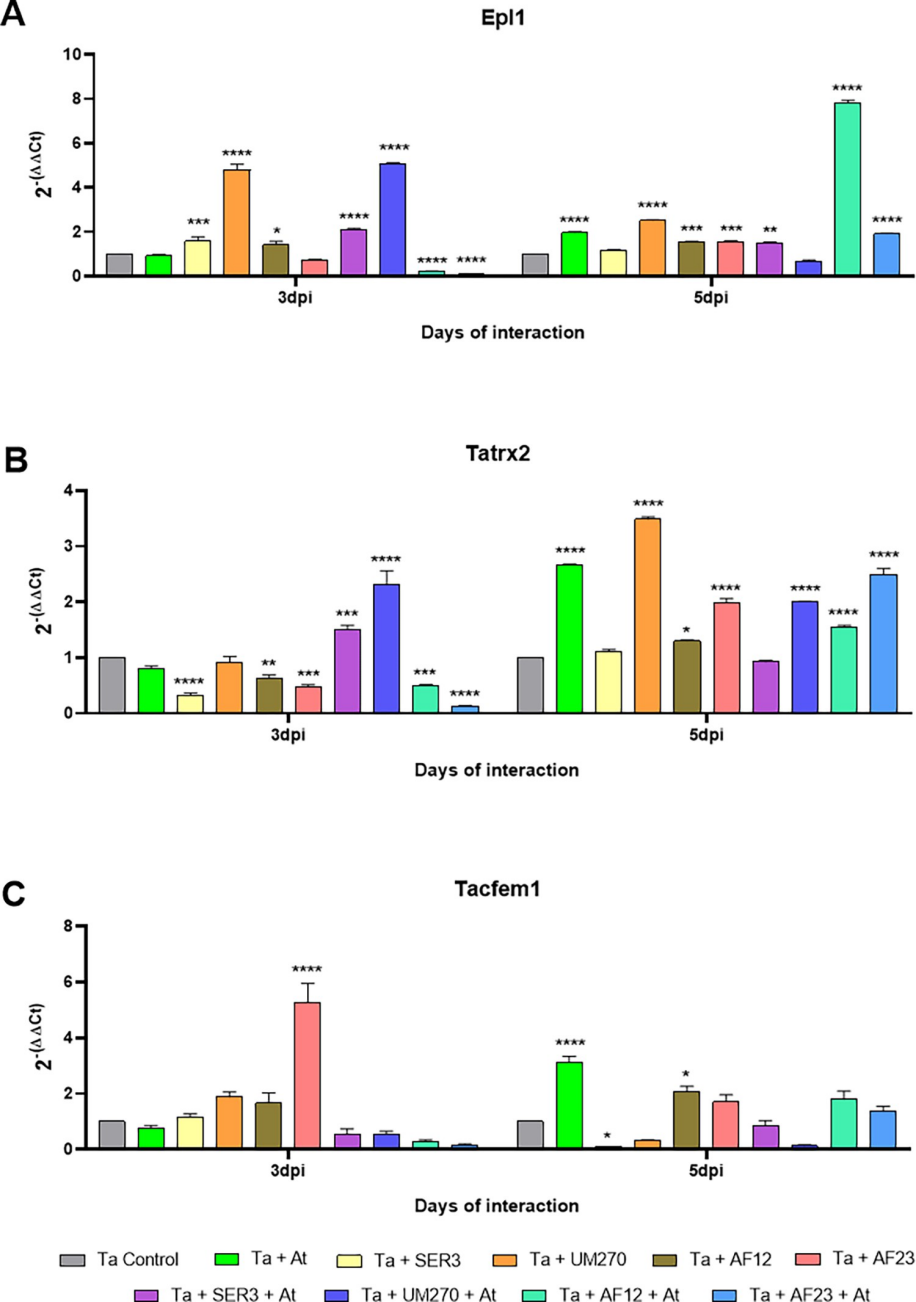
Relative gene expression of *Trichoderma* effectors in the beneficial interaction with the plant. Gene expression of *T*. *atroviride’*s effector coding genes *epl1* (A), *tatrx2* (B) and *tacfem1* (C) in the beneficial interaction with the plant At and PGPBs. Two-way ANOVA with Dunnet *post hoc* test of mean comparison, *p<0.05, **p<0.01, ***p<0.001, ****p<0.0001 compared to control conditions (fungus growing alone). At, *A*. *thaliana;* Ta, *T*. *atroviride*; SER3, *R*. *badensis* SER3; UM270, *P*. *fluorescens* UM270; AF12, *B*. *velezensis* AF12; AF23, *B*. *halotolerans* AF23.

Epl1 from *T*. *atroviride* is one of the best-known effectors of this fungus and is involved in conferring resistance to fungal and bacterial pathogens in plants, such as *A*. *thaliana* [22] and tomato [11]. At the 3^rd^ day of interaction (Fig 6A), *epl1* is downregulated in the presence of the plant and AF12 (0.23 ± 0.01; *p<*0.001) and in the presence of the plant and AF23 (0.10 ± 0.01; *p<*0.001). At this time of the interaction, the expression of *epl1* was not statistically different from the control in the presence of only *A*. *thaliana* or AF23 (*p>*0.05), but it was upregulated in the presence of SER3 (1.59 ± 0.19; *p<*0.001), AF12 (1.43 ± 0.14; *p<*0.05), and SER3+At (2.11 ± 0.06; *p<*0.001), and its expression was greater in the presence of UM270 and UM270+At (4.80 ± 0.25, 5.10 ± 0.03 fold increase, respectively; *p<*0.001) compared to the fungus growing alone. On the 5^th^ day of interaction, the expression of *epl1* was not statistically different compared to the control conditions in the presence of SER3 and UM270+At (*p>*0.05). The expression of *epl1* was upregulated during the interaction with At, UM270 (1.97 ± 0.05–2.52 ± 0.003 fold increase respectively; *p<*0.0001), AF12, and AF23 (1.55 ± 0.03–1.55 ± 0.05 fold increase respectively; *p<*0.001), and it was also upregulated in the presence of SER3+At (1.51 ± 0.04; *p<*0.01), AF12+At, and AF23+At (7.83 ± 0.11, 1.92 ± 0.06 fold increase respectively; *p<*0.0001), compared to the fungus growing alone. These results indicate that Epl1 may be involved in the interaction not only with plants such as *A*. *thaliana* but also during the interaction of *T*. *atroviride* with other beneficial microorganisms, such as the plant growth–promoting bacteria used in this study.

The gene *tatrx2* is induced in the presence of *A*. *thaliana* [23], and thioredoxins from *Glycine max* are required for nodule formation induced by Rhizobium [67]. In this work, at the 3^rd^ day post inoculation (Fig 6B), during the interaction with At and with UM270, *tatrx2* expression is not statistically different from the control (*p>*0.05), and its expression is downregulated compared to the control, in the presence of SER3 (0.32 ± 0.04; *p<*0.001), AF12 (0.63 ± 0.06; *p<*0.01), AF23 (0.47 ± 0.04; *p<*0.001), AF12+At (0.51 ± 0.01; *p<*0.01) and AF23+At (0.14 ± 0.01; *p<*0.001). Nonetheless, *tatrx2* was upregulated in the presence of SER3+At (1.52 ± 0.07 fold increase; *p<*0.001) and UM270+At (2.31 ± 0.23 fold increase; *p<*0.001) compared to the fungus growing alone. On the 5^th^ day of interaction (Fig 6B), the expression of *tatrx2* was not statistically different from the control conditions in the presence of SER3 and SER3+At (*p>*0.05), but its expression was upregulated in all the other experimental conditions. The treatments that increase *tatrx2*’s expression the most are At, UM270 and AF23+At (2.67 ± 0.01, 3.49 ± 0.04, 2.49 ± 0.11 fold increase respectively; *p<*0.0001), in comparison to the fungus growing alone. These results suggest that Tatrx2 may play a role in the interactions of *T*. *atroviride* with other beneficial microbes and beneficial microbes, especially *P*. *fluorescens* UM270.

Tacfem1 is a membrane receptor of *T*. *atroviride*, and we have previously demonstrated that the expression of *tacfem1* is induced during interaction with *A*. *thaliana* [23]. In our experimental conditions, on the 3^rd^ day of interaction with PGPBs and the plant *A*. *thaliana* (Fig 6C), we found no statistically significant differences in the expression of *tacfem1* in most of the treatments, compared to the fungus growing alone (*p>*0.05), and only in the presence of AF23, this gene was upregulated by 5.28 ± 0.67 fold (*p<*0.001) compared to the corresponding control. At the 5^th^ day of interaction, in the presence of SER3, *tacfem1* is downregulated compared to the control (0.09 ± 0.02; *p<*0.05), but this gene is upregulated in the presence of At (3.12 ± 0.22 fold increase; *p<*0.001) and AF12 (2.06 ± 0.20 fold increase; *p<*0.05) compared to the fungus growing alone. We found no significant differences compared to the control (*p>*0.05) in the expression of *tacfem1*. These results indicate that Tacfem1 may only be involved in the presence of some PGPBs used in this experiment and the plant, such as *B*. *halotolerans* AF23 and *B*. *velezensis* AF12.

Overall, the results from the expression of *T*. *atroviride* effector coding genes during the interaction with the PGPBs used and the plant *A*. *thaliana* show that *epl1* and *tatrx2* respond to the presence of the bacteria and the plant at different time points during the interactions, especially with the PGPB *P*. *fluorescens* UM270. While *tacfem1* respond mainly to the presence of the plant and bacteria *B*. *halotolerans* AF23 and *B*. *velezensis* AF12, suggesting the specificity of the effectors to the presence of different beneficial microorganisms.

#### Induction of plant defense systems by the consortia *T*. *atroviride–*PGPB

**Fig 7:**
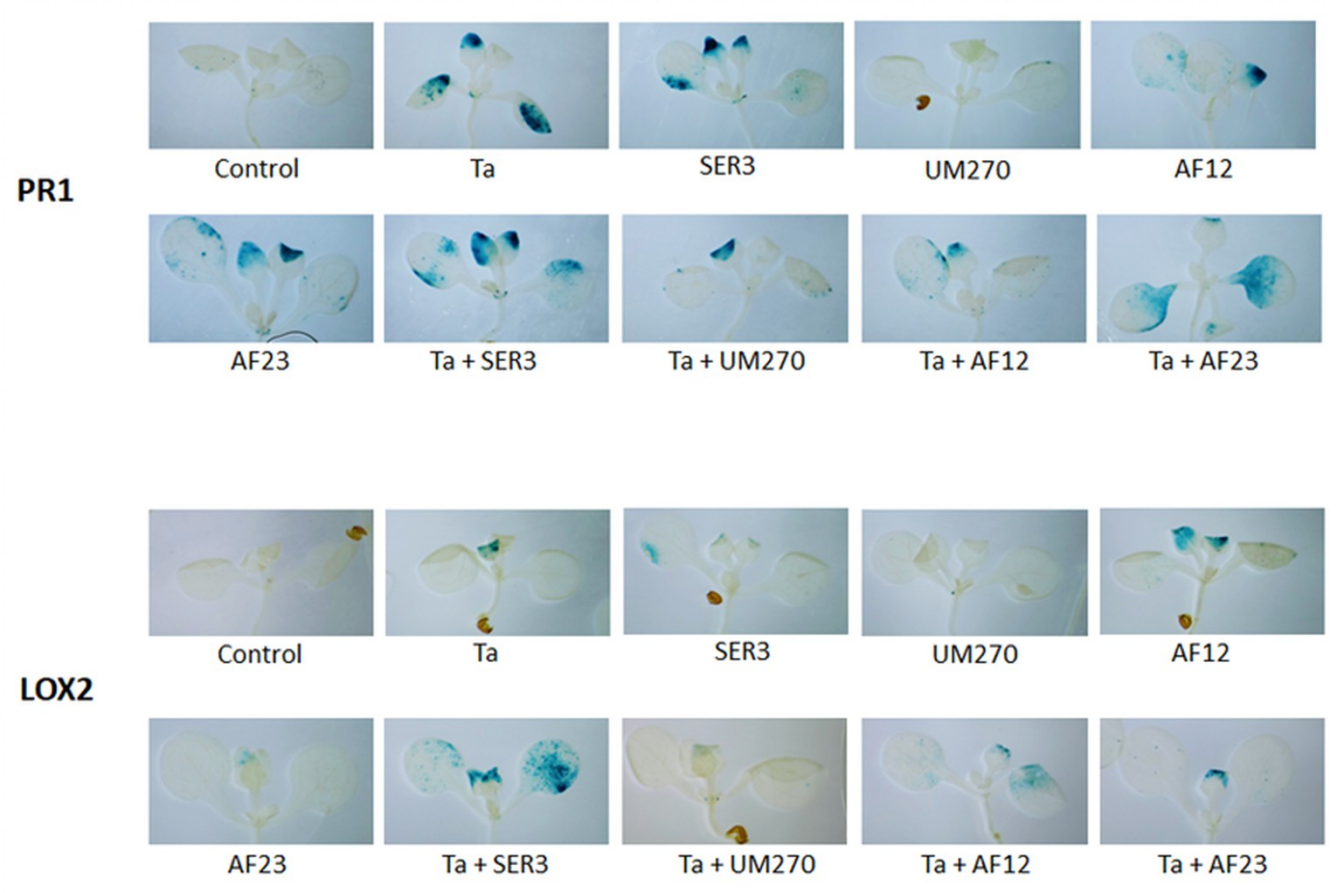
Induction of *A*. *thaliana* defense pathways. Induction of the *Arabidopsis* defense pathway mediated by salicylic acid (upper panel) and induction of the defense pathway mediated by jasmonic acid (downward panel) in the presence of the microorganisms alone or in consortium at the 5^th^ day of interaction. Presence and intensity of blue color indicate the accumulation of salicylic (PR1) and jasmonic (LOX2) acid. At, *A*. *thaliana;* Ta, *T*. *atroviride*; SER3, *R*. *badensis* SER3; UM270, *P*. *fluorescens* UM270; AF12, *B*. *velezensis* AF12; AF23, *B*. *halotolerans* AF23. The experiment was performed by triplicate with 10 plants in each replicate (n = 30). Photographs are representative of the experiment at the 5^th^ day post inoculation.

One of the indirect mechanisms of biocontrol shared by both *T*. *atroviride* and PGPB is the induction of plant SA-and JA–mediated defense systems, protecting plants from phytopathogen attack [68]. We evaluated the induction of plant defense systems using GUS transgenic lines of *A*. *thaliana*, PR1:GUS for SA-mediated defense responses, and LOX2:GUS for JA-mediated defense responses, the experiment was performed as indicated in the methodology section. Fig 7 shows the representative photographs of the plants at the end of the experiment (5^th^ dpi). As shown in Fig 7 upper panel, the presence of *T*. *atroviride* and PGPBs, except for UM270, induced SA accumulation compared to the plants growing alone, that is, control conditions. It is worth noting that the presence of UM270 does not induce SA accumulation, since the intensity of the blue color is almost the same as that of the control, and the intensity seems to diminish when the microorganisms are in consortium compared to when microorganisms are alone with the plant, suggesting that the presence of both beneficial microbes can induce SA accumulation, but to a lesser extent than the microorganisms alone.

As shown in Fig 7, JA accumulation is induced by the presence of all the microorganisms, alone or in combination, except for UM270, which does not induce JA accumulation compared to plants growing alone. During the interaction of *A*. *thaliana* with the consortia of microorganisms, the intensity of the blue color indicating accumulation of JA was greater than the intensity of the blue color when the plants were in the presence of the microbes alone, suggesting that the consortia are capable of inducing JA defense pathway to a greater extent than the microorganisms alone, being more noticeable in the consortium formed by *T*. *atroviride* and *R*. *badensis* SER3.

## Discussion

In the last decade, at least two or more species of beneficial microbes have been used as biocontrol agents and/or biofertilizers to improve plant growth and yield, showing promising results of their use compared to the use of microorganisms alone, in an attempt to mimic the conditions faced when in field applications, leading to tailoring microbial communities to suit specific requirements [69]. In this study, we aimed to establish consortia of two microorganisms between *T*. *atroviride* and four PGPBs, *P*. *fluorescens* UM270, *R*. *badensis* SER3, *B*. *velezensis* AF12, and *B*. *halotolerans* AF23, to determine if their combined effect resulted in better phytopathogen growth inhibition and ability to improve plant growth and defense systems, and whether *T*. *atroviride* effector coding genes may be involved in *T*. *atroviride–*PGPB interactions.

Regarding the growth inhibition capability of the consortia, although SER3 and Ta were the only efficient microorganisms on the 3^rd^ and 5^th^ days of confrontation with the pathogen *F*. *brachygibbosum*, on the 7^th^ day, the consortium Ta+SER3 was more effective at inhibiting the pathogen growth (78.96%), even better that the other consortia formed (Fig 2). We also noted that the growth inhibition ability of UM20, AF12, and AF23 increased when in combination with *T*. *atroviride*, compared to their ability when alone with the pathogen. These results are in accordance with those of several studies showing that *Trichoderma* improves its biocontrol capacity when combined with PGPBs [41].

For example, the combination of a commercial product made with *T*. *atroviride* SC1 and *B*. *subtilis* PTA-271 was able to protect *Vitis vinifera* plants from *Neofusicoccum parvum* disease; in direct confrontation *in vitro* with the pathogen, the combination of microorganisms showed better results than the microorganisms alone at inhibiting pathogen growth [70]. The combined inoculation of *Trichoderma* spp. and rhizobia in soil and *Vigna unguiculata* seeds resulted in less disease severity and symptoms caused by *R*. *solani* [71], showing greater biocontrol ability over the pathogen. A synthetic microbial community (SynCom) formed with several isolates of *Pseudomonas* spp., *Bacillus* spp., *Streptomyces* spp., and *Trichoderma* spp. showed effective biocontrol capacity against *F*. *oxysporum* f. sp. *cubense* (*Foc*) Tropical Race 4 and protected banana plants against the pathogen [72]. The results from our experiment clearly showed that the combination of *T*. *atroviride* and the PGPBs tested is a good option with great potential to be use as a biocontrol consortia instead of using only one microorganism, and that the interaction formed between *T*. *atroviride* and *R*. *badensis* SER3 showed promising results as a potential efficient biocontrol consortium against fungal pathogens. However, experiments to test the ability of the consortia formed in plant infection conditions need to be carried out to determine the biocontrol capacity of the microorganisms, which are part of the ongoing research.

Since both *T*. *atroviride* and PGPBs are known to be good biostimulants for plant growth, we tested the ability of the inoculation of microorganisms and their consortia on root system development and plant biomass of *A*. *thaliana* (Figs 3 and 4). Other studies have shown that the combination of *Trichoderma* spp. and other PGPBs, as well as other beneficial fungi such as mycorrhiza or other plant growth–promoting fungi (PGPF), have promising results as biostimulants in agriculture applications. For example, co-inoculation of *Trichoderma* spp. and rhizobia increased the biomass of *V*. *unguinculata* when inoculated in soil or by coating the seeds [71]. In addition, inoculation with *T*. *atroviride* MUCL45632 and the mycorrhizal fungus *Glomus intraradices* greatly improved shoot and root dry weights of tomato, zucchini, and lettuce plants, with better results than inoculation with the single organisms [73]. The consortium formed by *P*. *aeruginosa* DRB1 and *T*. *harzianum* CBF2 in the pesta granule formulation was able to improve plant growth of banana, and it also induced resistance against banana wilt caused by *F*. *oxysporum* fsp. *cubense* Tropical Race 4 (Foc-TR4), which is better than organisms alone [74,75]. According to our results, the consortium formed by *T*. *atroviride* and *P*. *fluorescens* UM270 could be used as a biostimulant, and this interaction yielded better results than the single inoculation of the microorganisms.

From the experiments on interactions between *T*. *atroviride* and the PGPBs tested, we observed that the effect of the consortia varied according to the PGPB in interaction with the fungus, and that the best biocontrol consortium was not the same as the best biostimulant consortium. Thus, we decided to analyze the relative expression patterns of some *T*. *atroviride* effector–coding genes previously identified and known to participate during interactions of this fungus with plants and/or phytopathogens, but their expression during interactions with PGPBs is unknown: *epl1* [11,21], *tacfem1* and *tatrx2* [23]. We analyzed their relative expression during confrontation with the phytopathogen *F*. *brachygibbosum* (Fig 5), in interaction with *A*. *thaliana* plants (Fig 6), and in the presence of each PGPB.

Cerato-platanins are a family of fungal small secreted cysteine-rich proteins (SSCPs), such as Epl1 from *T*. *atroviride*, and are widely known to induce plant systemic defense responses [21,76]. *epl1* is predominantly expressed during the development of *T*. *harzianum* and *T*. *guizhouense* [77], and *T*. *harzianum* secrets abundantly Epl1 in the presence of *F*. *solani* cell walls [78]. In our experiment on confrontation with *F*. *brachygibbosum*, *epl1* was not upregulated on the 3^rd^ day of interaction, but on days 5^th^ and 7^th^ days, *epl1* relative expression was induced compared to control conditions, except in the presence of *P*. *fluorescens* UM270 (Fig 5A). The best consortium to exert biocontrol over *F*. *brachygibbosum* in the *in vitro* experiment was Ta + SER3, and we observed that *epl1* was upregulated both in the presence of only the bacteria and in the presence of both the bacteria and the phytopathogen on days 5^th^ and 7^th^ days of confrontation, suggesting a possible role of Epl1 during the interaction with SER3. However, it may not be relevant to the biocontrol ability of Ta + SER3 since the expression of *epl1* was notably upregulated in the presence of *B*. *halotolerans* AF23 and in the consortium with these bacteria and the phytopathogen (Ta + AF23 + Fb), indicating that Epl1 may have a major role in interacting with *B*. *halotolerans* AF23 than with *R*. *badensis* SER3.

Some studies have shown the importance of Epl1 in mycoparasitic interactions of *Trichoderma* spp. Epl-1 from *T*. *harzianum* regulates virulence genes in *B*. *cinerea* during confrontation, which was proven when the *epl-1* knockout mutant from *T*. *harzianum* failed to downregulate the virulence genes, showing a role for Epl-1 in biocontrol over *B*. *cinerea* [79], but it appears to be not essential to exert biocontrol over other plant pathogens, such as *Sclerotinia sclerotiorum*, *R*. *solani* and *F*. *solani* [80]. Nonetheless, *epl1* from *T*. *gizhouense* and *T*. *harzianum* played a minor role during the interactions of these fungi with bacteria, such as *Escherichia coli* DH5α, *B*. *amyloliquefaciens* and *Ralstonia solanacearum*, and with the fungi *F*. *fujikuroi*, *R*. *solani*, *S*. *sclerotiorum* [77]. Our results show that *epl1* responds to the presence of the plant growth–promoting bacteria tested, suggesting that it may be important to *T*. *atroviride* during interactions with such microorganisms. However, more extensive research is needed to determine the role of *epl1* from *T*. *atroviride* during mycoparasitic interactions and in consortia with other beneficial microorganisms.

During interactions with plants, Epl1 from *T*. *asperellum* promotes the growth of *Populus davidiana* × *P*. *alba* var. *pyramidalis* (PdPap) seedlings and *epl1* expression is induced under different conditions, such as root and leaf powder from PdPap seedlings [60], indicating that it is also involved in plant growth promotion in addition to being a plant defense elicitor molecule [11,22]. Our results from the experiment with PGPBs and *A*. *thaliana* showed upregulation of *epl1* in the presence of the plant on the 5^th^ day of interaction, and in the presence of the PGPBs tested. It is worth noting that *epl1* was induced in the presence of UM270 and UM270 + At at the beginning of the interaction (Fig 6A), and on the 5^th^ day, it was upregulated in the presence of AF12 + At. Epl1 has been shown to be important in regulating ISR induction in tomato [11], protecting plants against different pathogens such as *P*. *syringae* pv. *Tomato*, *A*. *solani* and *B*. *cinerea*, its induction in the presence of PGPBs suggests the possibility that the consortium could induce plant defense systems.

Our results also indicate that Epl1 may be involved in the interaction not only with *A*. *thaliana* but also during the interaction of *T*. *atroviride* with other beneficial microorganisms, such as the PGPBs used in this study, and it responds to their presence. Thioredoxins have been studied because of their importance in biological processes, such as growth and oxidative stress tolerance in fungi [61–63], and as effectors in plant pathogens, such as the thioredoxin GpPDI1 from the plant parasitic nematode *Globodera pallida* [81]. We have previously reported that the gene *tatrx2* from *T*. *atroviride* codes for an effector protein within the thioredoxin family with a role in the interaction with *A*. *thaliana*, but it seemed not to be important during the confrontation with *R*. *solani* [23]; however, there is little information about *Trichoderma* thioredoxins during interactions with other organisms.

Our results from the relative expression of *tatrx2* during the mycoparasitic interactions and PGPBs showed that this gene is mainly upregulated at the early stage of the confrontation, and only in the presence of SER3, AF23, UM270 + Fb, and AF23 + Fb, but not in the presence of the other PGPBs (Fig 5B), suggesting that the protein encoded by this gene, unlike *epl1* which was upregulated during interaction with all PGPBs tested and at later stages of the interaction, may only play a role in interaction with some PGPBs, such as *R*. *badensis* SER3 and *B*. *halotolerans* AF23 at the beginning of the confrontations.

Thioredoxins are known to be important during nodule formation in *Rhizobium* and soy plants [67], and in our previous study, *tatrx2* was induced in *Arabidopsis* in contact with the plant roots [23]. In this study, *tatrx2* was also upregulated in the presence of the plant at the 5^th^ day of the interaction; however, *tatrx2* expression was downregulated in most treatments at the beginning of the interaction with the plant, but its expression increased by the 5^th^ day, especially in the presence of UM270, and also in the presence of AF12+AT and AF23+At, which were downregulated on the 3^rd^ day, indicating that this gene responds not only to the presence of *A*. *thaliana* but also to the presence of most of the PGPBs tested.

Both *epl1* and *tatrx2* are induced mainly when *T*. *atroviride* interacts with UM270, and the consortium formed by Ta + UM270 showed better results in promoting plant biomass and root system, which may indicate that the proteins encoded by these genes play a role in the interaction with *P*. *fluorescens* UM270 and *A*. *thaliana*. Common fungal extracellular membrane (CFEM) proteins are unique to fungi [65] and have been related mainly to pathogenicity in *Magnaporthe oryzae* [82] and *Puccinia striiformis* f. sp. *tritici* [83], for example, and several CFEM domain-containing proteins were found in the secretome of *Trichoderma* spp. in co-cultivation with *A*. *thaliana* [84]. In our previous work, we found that *tacfem1* was upregulated when confronted with the phytopathogen *R*. *solani* anastomosis group AG5 during contact and overgrowth of the pathogen, but not when confronted with the anastomosis group AG2, and it was upregulated in the interaction with *A*. *thaliana* on days 5^th^ and 7^th^ days [23], however, there is little information on CFEM proteins from *Trichoderma* spp. in interaction with other organisms, especially with PGPBs.

Our results showed that *tacfem1* was upregulated on the 3^rd^ day of confrontation in the presence of the pathogen Fb, and all PGPBs, except for SER3, but on the 5^th^ day of confrontation only in the presence of UM20 and AF23 + Fb, this gene was upregulated (Fig 5C). However, at the 7^th^ day it was strongly induced in the presence of Fb, SER3, and UM270, suggesting that Tacfem1 could be involved at the end of the confrontation, when *Trichoderma* overgrows the pathogen and is in contact with some of the PGPBs, *R*. *badensis* SER3, and *P*. *fluorescens* UM270.

In our experiment on the interaction with *Arabidopsis*, *tacfem1* was induced on the 3^rd^ day only in the presence of AF23, whereas on the 5^th^ day, it was upregulated in the presence of *Arabidopsis* and AF12, suggesting that Tacfem1 may only be involved in the presence of some of the PGPBs used in this work, such as *B*. *halotolerans* AF23 and *B*. *velezensis* AF12. It is worth mentioning that the expression pattern of each of the *T*. *atroviride* effectors tested in this work is different according to the beneficial microbes the fungus is with, and if both are in the presence of a phytopathogen or a plant, this suggests a differential response or specificity of each gene according to the type of microorganism and interaction *T*. *atroviride* is with.

As mentioned previously, both *T*. *atroviride* and PGPB are capable of inducing plant SA-and JA–mediated defense systems, leaving plants resistant to pathogen attack [68]. *Trichoderma* can modulate SA and JA accumulation in plants, so it can colonize its host and establish the interaction, changing the phytohormone balance by increasing SA signaling and diminishing JA signaling [85,86], which is in accordance with our results, which show that *T*. *atroviride* induces SA accumulation and diminishes JA accumulation in the transgenic lines used (Fig 7). The consortia also showed a similar pattern of modulating SA- and JA-mediated defense responses, suggesting that the presence of both beneficial microbes is capable of modulating phytohormone balance in *Arabidopsis*. These results suggest that different combinations of microorganisms could have different results and diverse applications regarding the protection of the plant; thus, one could modulate the plant response to pathogen attack according to the consortium used, inducing one or the other defense pathway. Nonetheless, confirmation of the induction of both SA and JA defense pathways using gene expression or SA and JA *Arabidopsis* mutants is relevant to support our findings and are part of the ongoing research.

Taken together, biocontrol agents (BCAs) should be able to antagonize phytopathogens or induce resistance in their plant hosts, but also should be able to promote plant growth and yield [74], so the consortium formed by two or more BCAs, ideally, should be better than the microorganisms alone, in terms of both plant growth and protection against pathogens.

## Conclusion

*Trichoderma* has been widely regarded as an excellent partner because of its beneficial multi-kingdom interactions [87]. Similarly, PGPBs are also important actors in the plant microbiome, improving plant growth and health [1]. As part of the plant microbiome, the presence of *Trichoderma* and PGPBs has its perks, such as plant growth, promotion, protection against plant pathogens, and induction of plant defense systems. However, to exert such benefits, these microorganisms must establish a molecular dialogue with the plant, the pathogens, and each other. Here, we show that the consortia of *T*. *atroviride* and PGPBs have promising uses as biocontrol agents and plant biostimulants, and effector-like proteins from *T*. *atroviride* might be involved in the relationships with the plant and other beneficial bacteria.

## Supporting information

S1 Fig Co-inoculation conditions of *T*. *atroviride* and PGPBs. Each PGPB was streaked in a cross shape along the Petri plate containing PDA medium, forming four quadrants; in each quadrant, plugs of actively growing mycelia (upper-left and down-right quadrants) and 1x10^6^ conidia from *T*. *atroviride* (upper right and down left quadrants) were inoculated. Ta, *T*. *atroviride*; SER3, *R*. *badnesis* SER3; UM270, *P*. *fluorescens* UM270; AF12, *B*. *velezensis* AF12; AF23, *B*. *halotolerans* AF23. Experiment was performed with three replicates.
(TIF)

S1 Table List of primers. The list of primers used in this study are shown and described in the table.
(DOCX)
