# Supplementary figures and images for: Plant growth-promoting bacteria potentiate antifungal and plant-beneficial responses of Trichoderma atroviride by upregulating its effector functions
Source: PLoS One. 2024 Mar 22;19(3):e0301139. doi: 10.1371/journal.pone.0301139 (PMC10959389; doi:10.1371/journal.pone.0301139)

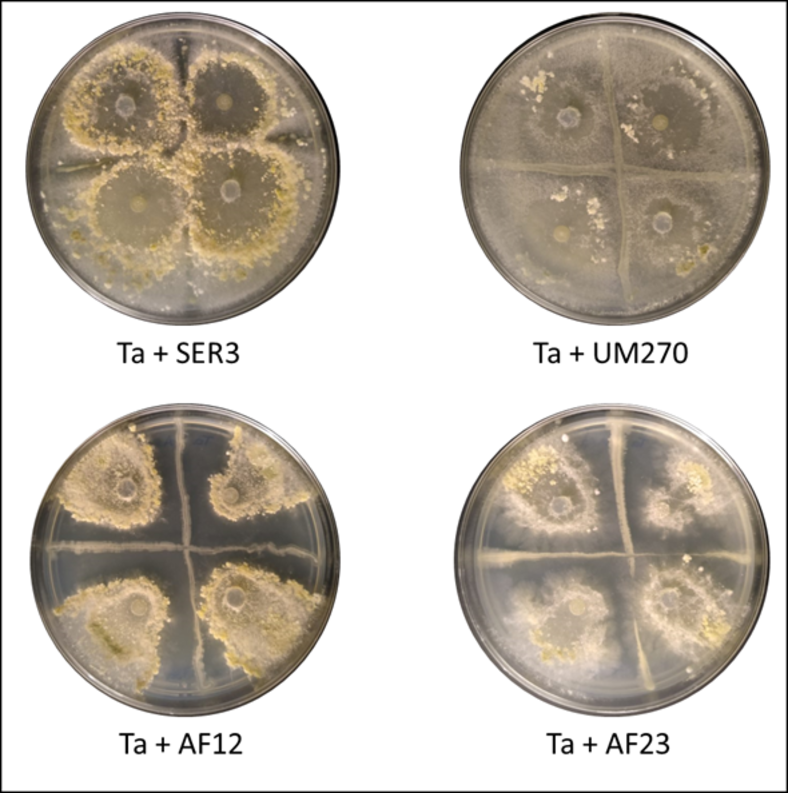

Supplement: S1 Fig — Each PGPB was streaked in a cross shape along the Petri plate containing PDA medium, forming four quadrants; in each quadrant, plugs of actively growing mycelia (upper-left and down-right quadrants) and 1x10^6^ conidia from T. atroviride (upper right and down left quadrants) were inoculated. Ta, T. atroviride; SER3, R. badnesis SER3; UM270, P. fluorescens UM270; AF12, B. velezensis AF12; AF23, B. halotolerans AF23. Experiment was performed with three replicates. (TIF) [file pone.0301139.s001.tif]
