# Supplementary material for: Plant growth-promoting bacteria potentiate antifungal and plant-beneficial responses of Trichoderma atroviride by upregulating its effector functions
Source: PLoS One. 2024 Mar 22;19(3):e0301139. doi: 10.1371/journal.pone.0301139 (PMC10959389; doi:10.1371/journal.pone.0301139)
Supplement: S1 Table — The list of primers used in this study are shown and described in the table. (DOCX) [file pone.0301139.s002.docx]

**Supporting Information**

**S1 Table.**

| **LIST OF PRIMERS USED FOR THIS STUDY** | | | |
| --- | --- | --- | --- |
| **Name** | **Sequence 5'→3'** | **Use** | **Fragment** |
| TaTrx2AS | AGGGAACTTGTCGGTCTTGAGGTT | qRT-PCR for gene *tatrx2* | 178bp |
| TaTrx2S2TR | TGCCGGTATCCCTTTGGCTTACAT |  |  |
| TaCFEM1AS | CATCGGCACCACAAGCAGCAATTA | qRT-PCR for gene *tacfem1* | 96bp |
| TaCFEM1S2TR | CAGACACTGACTTGGCATGCGTTT |  |  |
| Epl1F | GCCGCAGTCTCTGCTGATAC | qRT-PCR for gene *epl1* | 124bp |
| Epl1R | GGGTTTGCCAGTGGTATCT |  |  |
| GPD-F | GCTGCCGATGGTGAGCTCAAGGG | qRT-PCR for the housekeeping gene *gpd* | 189bp |
| GPD-R | GAGGTCGAGGACACGGCGGGA |  |  |
